# Diastereoselective synthesis of some novel benzopyranopyridine derivatives

**DOI:** 10.1186/1860-5397-2-25

**Published:** 2006-12-07

**Authors:** Pradeep K Mohakhud, Sujeet M R Kumar, Vasanth M R Kumar, Ravi M R Kumar, Moses D R Babu, Vyas D R K, Om D R Reddy

**Affiliations:** 1 Technology Development Centre, Dr Reddy's Research Laboratories Ltd., Bollaram Road, Miyapur, Hyderabad-500 050, India

## Abstract

**Background:**

The formation of novel *N*-substituted-1,2,3,4-tetrahydro[1,3]-dioxolo-[6,7]-5*H*-[1]benzopyrano [3,4-*c*]pyridines were observed unexpectedly during the acid-mediated ketal removal of ethylenedioxy ketal protected 4-piperidones. The literature revealed that benzopyranopyridine derivatives are of scientific interest and some exhibit interesting biological activities. Diastereomeric resolution was utilized to isolate optically pure chiral molecules.

**Results:**

The acid catalyzed deprotection of *N*-substituted-4,4-ethylenedioxy-3- [(1,3-benzodioxol-5-yloxy)methyl]piperidines, prepared by condensation of the corresponding phenols and mesylate derivatives, unexpectedly resulted in cyclodehydration leading to new benzopyrano derivatives, *N*-substituted-1,2,3,4-tetrahydro[1,3]-dioxolo-[6,7]-5*H*-[1]benzopyrano [3,4-*c*]pyridines. The process involves the deprotection of the carbonyl protecting group, and then the cyclization reaction occurs followed by dehydration to give the final product.

These *N*-substituted-1,2,3,4-tetrahydro[1,3]-dioxolo-[6,7]-5*H*-[1]benzopyrano [3,4-*c*] pyridines were dealkylated giving the corresponding *N*-unsubstituted derivatives. The *cis*-1,3,4,4a,5,10b-hexahydro-[6,7]-2*H*-[1]benzopyrano [3,4-*c*]pyridine derivative was also obtained from the *N*-benzylated-1,2,3,4-tetrahydro[1,3]-dioxolo-[6,7]-5*H*-[1]benzopyrano [3,4-*c*]pyridine *via* catalytic hydrogenation. The resolution of the enantiomers was carried out using *D*-(-)-mandelic acid as chiral reagent. The absolute configuration of the *S*,*S*-mandelate salt derivative was determined by X-ray crystallographic analysis.

**Conclusion:**

The approach led to the construction of *N*-substituted-1,2,3,4-tetrahydro[1,3]-dioxolo-[6,7]-5*H*-[1]benzopyrano [3,4-*c*] pyridines ring systems involving the one-pot deprotection, cyclization and dehydration of *N*-substituted-4,4-ethylenedioxy-3- [(1,3-benzodioxol-5-yloxy)methyl]piperidines. The hydrogenation of the *N*-benzylated benzopyrano [3,4-*c*]pyridine derivative followed by resolution led to the formation of a new compound.

## Introduction

The present paper describes the study of a novel synthesis of benzopyranopyridine derivatives obtained by the unexpected cyclization of 3-substituted aryloxy methyl-4,4-ethylenedioxy-*N*-substituted piperidine derivatives (**4a**, **4b**). This was discovered whilst attempting the deprotection of the ethylenedioxy ketal at the 4-position to form intermediate **4c** for the preparation of the antipsychotic drug Paroxetine. [1] This compound was characterized and the literature search revealed that such a compound has not been synthesized by this strategy.

It was also found in the literature that various nitrogenous analogs of tetrahydro cannabinole derivatives like 1,2,3,4-tetrahydro-5*H*-[1]-benzopyrano[3,4]pyridine-5-one (**A**) were synthesized by the Pechman condensation [2] and are potent bronchodilators, [3] which have the potential to be useful in the treatment of asthma and bronchitis. Furthermore, it is reported that benzopyrano derivatives of type **B**, which exhibit significant CNS depressant and hypotensive activity [4] were prepared from derivatives of compound **A**.

**Figure 1:**
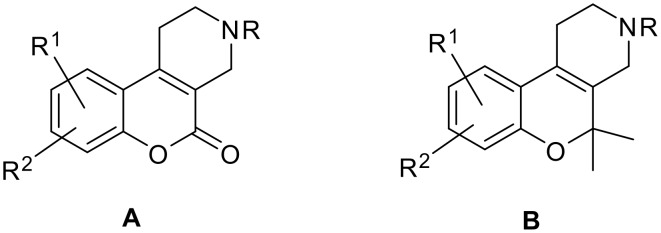
General structures **A** & **B**, Tetrahydro cannabinole derivatives.

A recent patent [5] relates to a compound of general structure **C**, and the pharmaceutically accepted salts, which exhibited α-1 adrenergic antagonist behavior and are useful in the treatment of benign prostatic hyperplasia (BPH) and other urological diseases. Taking in to account the available medicinal chemistry data, molecules of types **C** and **D** are interesting synthetic targets, especially as they have a wide spectrum of chemical reactivity as well as diverse and marked biological activities, which are useful for drug discovery research.

**Figure 2:**
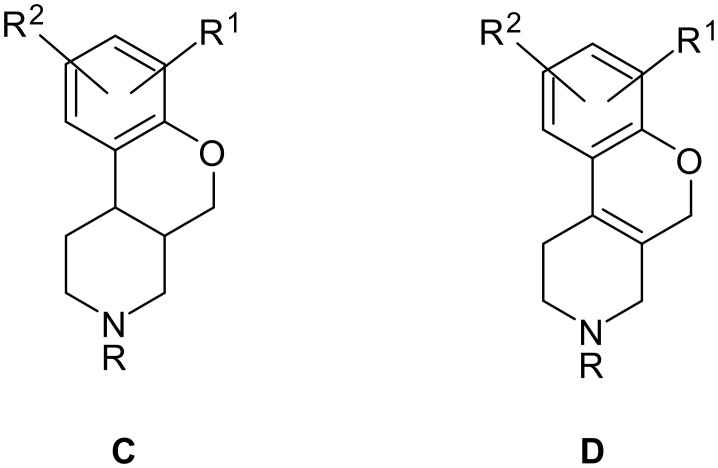
General structures **C** & **D**, Benzopyrano pyridine derivatives.

To the best of our knowledge, compounds with the general structure **D** (with no substitution at C-5) have not been reported. Compounds of type **C** have been reported with the "*trans*" configuration, [5] while we report the synthesis of compounds with the *cis* configuration by a different synthetic approach. The reported synthesis [5] for the *trans* compound involved reaction of ethyl 2-methoxy-6-methoxymethyl cinnamate with ethyl *N*-benzylamidomalonate followed by reduction with LiAlH_4_. Subsequent conversion of the hydroxymethyl to a leaving group and then intramolecular cyclization, followed by debenzylation, furnished the *racemic trans* 10-methoxy-1,3,4,4a,5,10b-hexahydro-2*H*-[1]-benzopyrano [3,4-*c*]pyridine.

## Results and discussion

**Scheme 1:**
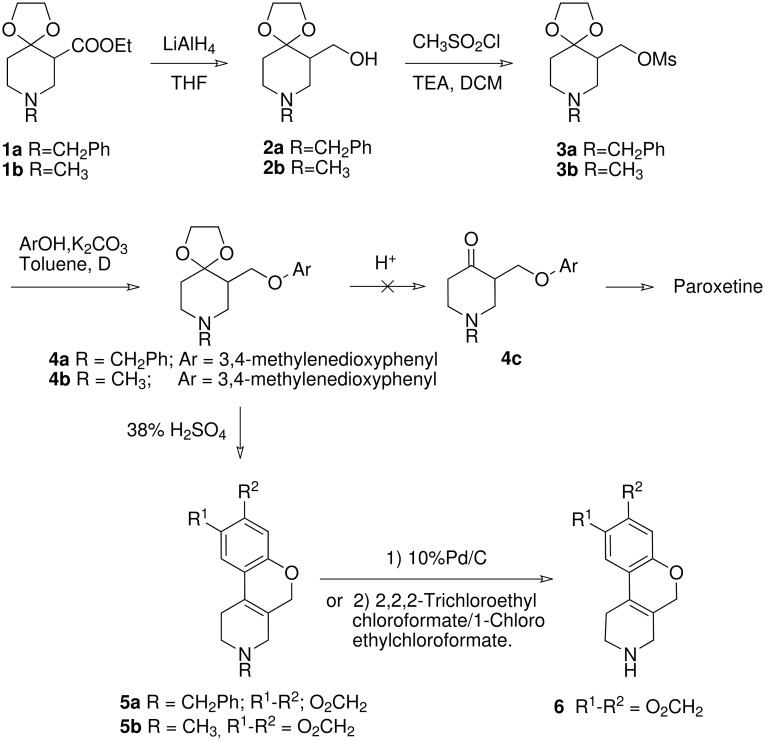
Synthetic sequence to tetrahydropyridine compounds.

**Scheme 2:**
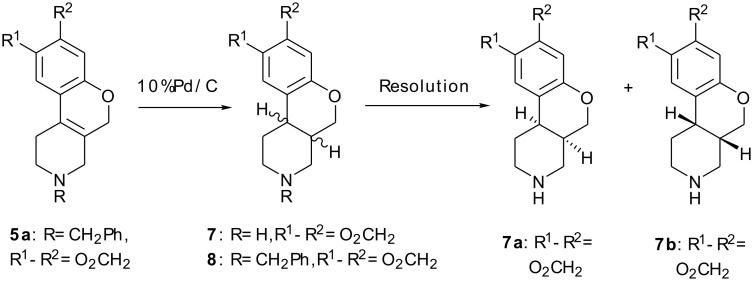
Resolution of enantiomers using D(-) mandelic acid.

The present study relates to the synthesis of new molecules of type **C** with *cis* configuration (**7a**, **7b**, **8**) as shown in Scheme 2 and new molecules of type **D** (**5a**, **5b**, **6**) as shown in Scheme 1. Also the intermediates **3a**, **3b**, **4a**, **4b** involved during the synthesis of these new molecules are not reported in the literature but are explained below (For full experimental data, see also: Supporting Information File 1).

### Serendipitous synthesis of 1,2,3,4-tetrahydro[1,3]-dioxolo-[6,7]-5*H*-[1] benzopyrano [3,4-*c*] pyridine skeleton

The synthesis of these compounds involved the reduction of *N*-substituted 3-carbethoxy-4,4-ethylenedioxy piperidines **1a** and **1b**, [6–7] using lithium aluminum hydride to form **2a** and **2b**. The ketones in **2a** and **2b** were deprotected in acidic conditions, but the required *N*-substituted-3-hydroxymethyl-4-piperidones were not isolated. From the literature, [6] it was found that heating **2a** with 20% hydrochloric acid resulted in dimerization leading to 2,8-dibenzyldecahydro-2*H*,5a*H*-4a,9a-epoxydipyrido [4,3-b:3'4'-*f*]oxepin-5a-ol. Accordingly, alcohols **2a** and **2b** were treated with methanesulfonylchloride, in the presence of base, which provided the required 3-mesylate derivatives **3a** and **3b**. Mesylates **3a** and **3b** were condensed with 3,4-methylenedioxyphenol in the presence of base and solvent to give ethers **4a** and **4b**. These ethers, on treatment with acid, underwent unexpected cyclization to give rise to **5a** and **5b**, instead of the desired ketone **4c**. A proposed mechanism for the cyclodehydration leading to the cyclized product is discussed below. The cyclized product **5b**, was dealkylated using chloroformates by two different methods to yield **6** as a free base. Also compound **6** was obtained by hydrogenolysis of **5a** (Scheme 1).

The reaction mechanism is suggested to follow a ketal deprotection sequence to arrive at the 4-piperidone derivative, which is protonated and reacts with the aromatic ring to afford the six member heterocycle. Deprotonation restores the aromatic ring and subsequent elimination of water results in the stabilized cyclized product **5b**. The structural elucidation of **5b** confirmed the benzopyranopyridine skeleton (see below).

**Figure 3:**
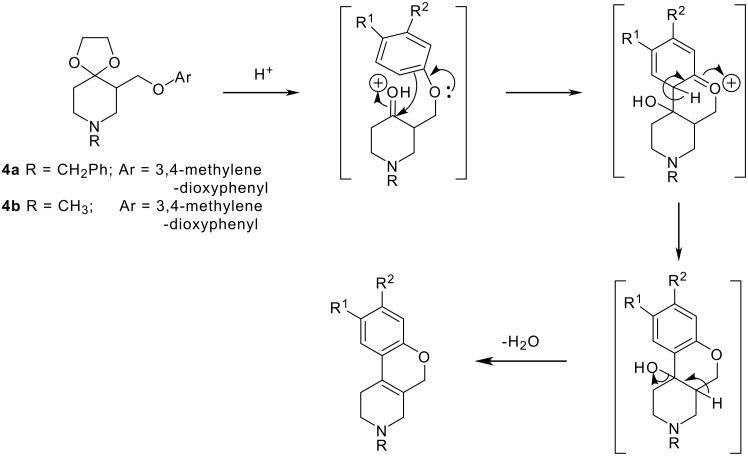
Proposed mechanism of tetrahydropyridine formation, Proposed mechanism.

**Figure 4:**
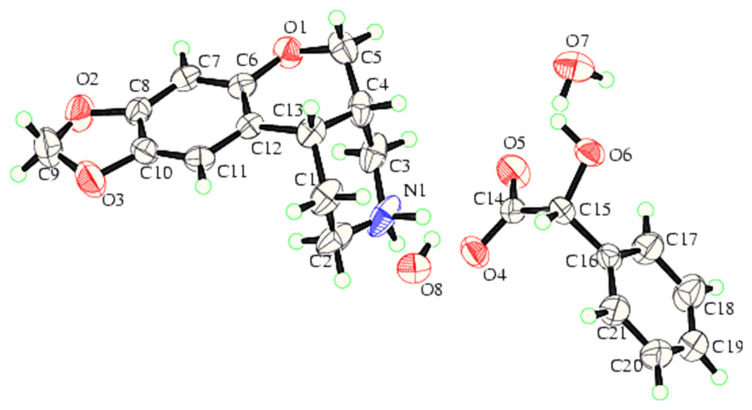
Molecular structure of the mandalate salt.

The saturated product *cis*-1,3,4,4a,5,10b-hexahydro-[6,7]-2*H*-[1]-benzopyrano- [3,4-*c*]pyridine **7** (skeleton C) was made by hydrogenation of *N*-benzylated benzopyranopyridine derivative **5a** using 10% palladium on charcoal in a methanol-HCl mixture (Scheme 2). Initial hydrogenation experiments provided *N*-benzyl saturated pyridine derivative **8** and *N*-unsubstituted unsaturated product **6**. After extended reaction time, debenzylated saturated product **7** was formed with the *cis* configuration. Compound **6** can be obtained by terminating the reaction after 4–6 hours followed by chromatographic purification of the crude. The enantiomers were separated by diastereomeric resolution using *D*-(-)-mandelic acid as the chiral agent. The absolute configuration of the mandelate salt of **7b** was determined as S,S by X-ray diffraction analysis (Figure 4) (For details see also: Figure 4 and Supporting Information File 1).

### Structure elucidation of 5b

The mass spectrum of **5b** displayed a molecular ion at m/z 245 corresponding to the molecular formula C_14_H_15_NO_3_. Interestingly the molecular ion is 18 amu less than the expected structure **4c** (R = Me). The ^1^H NMR data in the aromatic region showed only two sharp singlets at 6.4 and 6.6 ppm contrary to the three expected signals i.e. one singlet and two doublet signals for the structure **4c** (R = Me). The missing aromatic signal in structure **5b** could have been involved in the bond formation. It is also interesting to note that in the DEPT experiment, the only aliphatic methine signal expected for the structure **4c** (R = Me) is not present. The absence of characteristic ketonic absorption in IR and absence of quaternary signal beyond 150 ppm in ^13^C NMR indicated the absence of a ketonic functionality in structure **5b**, which is further confirmed by the NOESY experiment, and the through space interactions (nOe's) are shown in **5b**-nOe. The formation of this new skeletal system has been confirmed beyond doubt by the single crystal X-ray studies of **7b**. The compound **7** is formed from the hydrogenation of **5a** and **6**.

**Figure 5:**
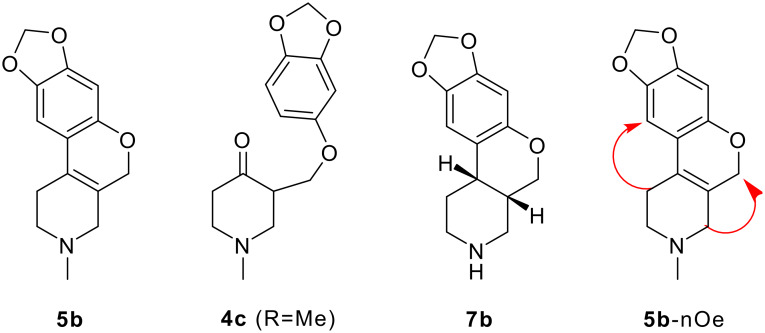
Structural elucidation.

## Conclusion

In summary, the synthesis of novel *cis*-1,3,4,4a,5,10b-hexahydro-[6,7]-2*H*-[1]benzopyrano [3,4-*c*]pyridine **7** is achieved using a different synthetic route than that reported for a similar ring structure with a *trans* configuration. This paper reports the formation of **7** *via* key intermediates **5a** and **5b**. It is hypothesized that the presence of the activated aromatic ring facilitates the cyclodehydration in acidic medium leading to the benzopyranopyridine derivative. The saturated *cis* derivative **7** is successfully resolved to get the individual enantiomers **7a** and **7b**. The absolute configuration is determined by single crystal X-ray analysis of the mandelate salt.

## Supporting Information

File 1 Supplementary experimental data. The file contains all experimental procedure and analytical data belonging to the compounds described in the article.
