# Supplementary material for: Diastereoselective synthesis of some novel benzopyranopyridine derivatives
Source: Beilstein J Org Chem. 2006 Dec 7;2:25. doi: 10.1186/1860-5397-2-25 (PMC1779792; doi:10.1186/1860-5397-2-25)
Supplement: File 1 — Supplementary experimental data. The file contains all experimental procedure and analytical data belonging to the compounds described in the article. [file Beilstein_J_Org_Chem-02-25-s001.doc]

**Supplementary experimental data**

**Experimental details:**

Melting points were taken in an open capillary tube on Buchi B 540 apparatus and are uncorrected. The ^1^H NMR spectra were recorded with 200 MHz Varian Gemini spectrometer with TMS as an internal standard. Infrared spectra were recorded using a Perkin-Elmer model 1640 apparatus. Mass spectra were recorded on an MS Engine Hewlett-Packard model 5989.

**1-Benzyl -4,4-ethylenedioxy-3-methylsulfonyloxymethylpiperidine (3a)**

To a mixture of alcohol **2a** (123.6g, 0.47moles) in dichloromethane (620mL) and triethylamine (52.3g, 0.518moles) at 0-5 ^0^C, methane sulfonyl chloride (58.4g, 0.51moles) was added and stirred at the same temperature. After 3h water (200.0mL) was added to the reaction mixture and the organic layer was separated, dried (MgSO_4_). Solvent evaporated in *vacuo* and the crude is recrystallized from diisopropyl ether (250.0mL) to give the title compound **3a** (129.8 g, 81%) as a light yellow solid, mp : 59-61 ^0^C, IR (νmax, KBr): 3444, 2923, 2834, 1356, 1175, 958, 750, 528 cm^-1^, ^1^H NMR (CDCl_3_): δ7.2 (5H, s, Ph), 4.42-4.2 (2H, m, 2xH-3a), 3.85 (4H, s, OCH_2_CH_2_O), 3.6(1 H, d, J 13.0 Hz, CH_a_H_b_Ph), 3.45(1 H, d, J 13.0 Hz, CH_a_H_b_Ph) , 2.82 (3H, s, CH_3_), 2.8-2.25 (4H, m, 2xH-6 + 2x H-2), 2.25-2.15 (1H, m, H-3), 1.9-1.8 (2H, m, 2xH-5) MS (m/z) : 341 (M^+^), 262, 246, 91.

**1-Methyl-4,4-ethylenedioxy-3-methylsulfonyloxymethylpiperidine (3b)**

To a mixture of alcohol **2b** (87.9g, 0.47moles) in dichloromethane (440 mL) and triethylamine (52.3g, 0.518moles) at 0-5^0^C, methane sulfonyl chloride (58.4g, 0.51moles) was added and stirred at the same temperature. After 3h water (200.0mL) was added to the reaction mixture and the organic layer was separated, dried (MgSO_4_). Solvent evaporated in *vacuo* and the crude is recrystallized from diisopropyl ether (180.0mL) to give the title compound **3b** (99.60 g, 80%) as off white solid, mp : 73-74 °C, IR (νmax, KBr): 3441, 2959, 2802, 1344, 1168, 956, 853, 528 cm^-1^, ^1^ HNMR (CDCl_3_): δ 4.4, (1 H, dd, J 10.0, 3.0 Hz, H-3a), 4.3 (1H, m, H-3a), 3.95 (4H, br, OCH_2_CH_2_O), 3.0 (3H, s, SO_2_CH_3_), 2.8 (1H, m, H-2), 2.6 (1H, m, H-2), 2.4-2.15 (6H, br, N-CH_3_ + 2 x H-6 + H-3), 1.8-1.65 (2H, br, 2 x H-5), ^13^C NMR (CDCl_3_): δ 33.81 (C-5), 37.14 (SO_2_CH_3_), 43.34 (C-3), 45.74 (N-CH_3_), 53.15 (C-6), 55.64 (C-2), 64.49 (OCH_2_CH_2_O), 68.02 (CH_2_OSO_2_), 106.98 (C-4), MS (m/z): 265 (M^+^) 186, 170, 91.

**1-Benzyl-4,4-ethylenedioxy-3-[(1,3-benzodioxol-5-yloxy)methyl]piperidine (4a)**

The mesylate derivative **3a** (59.67g, 0.175 moles) was added to a mixture of toluene (500.0mL), and 3,4-methylenedioxy phenol (24.6g, 0.178 moles) and powdered potassium carbonate (60.6g, 0.439 moles) and refluxed for 20-24 h. Water (1.0L) was added to the cooled reaction mass. The organic layer was separated and the aqueous layer was extracted with toluene (250.0mL). Both the combined organic layer was washed with 10% sodium hydroxide solution (250.0mLx 2), brine (250.0mLx 2). The organic layer was evaporated in *vacuo* to give the title compound **4a** (65.7g, 98%) as brown colored oil. IR (νmax, KBr): 2952, 2885, 2811, 1631, 1488, 1185, 1099, 1038, 925, 746, 700 cm^-1^, ^1^H NMR (CDCl_3_): δ 7.5 (5H, s, Ph), 6.7 (1H, d, J 9 Hz, H-5’), 6.5 (1H, d, J 3 Hz, H-2’), 6.3 (1H, dd, J 9.0, 3.0 Hz, H-6’), 5.9 (2H, s, O_2_CH_2_), 3.95 (6H, s, 2x H-3a+OCH_2_CH_2_O), 3.58 (1 H, d, J 13.0 Hz, CH_a_H_b_Ph), 3.46 (1 H, d, J 13.0 Hz, CH_a_H_b_Ph), 2.9 (1H, m, H-2), 2.7-2.3 (4H, m, H-2+2xH-6+H-3), 1.7 (2H, m, H-5),

^13^C NMR (CDCl_3_): δ 33.52 (C-5), 43.18 (C-3), 50.38 (C-6), 53.60 (C-2), 61.93 (N-CH_2_), 63.96 (OCH_2_CH_2_O), 64.01 (OCH_2_CH_2_O), 66.22 (CH_2_O), 97.73 (C-5’), 100.52 (O_2_CH_2_), 105.41(C-2’), 107.38 (C-6’), 107.53 (C-4), 126.43 (C-4’’),127.70 (C-2’’,C-6’’), 128.36 C-3’’,C-5’’), 138.17 (C-1’’) ,141.04 (C-1’), 147.71 (C-4’), 154.16 (C-3’), MS (m/z): 383 (M+), 246, 91.

**1-Methyl-4,4,-ethylenedioxy-3-[(1,3-benzodioxol-5-yloxy)methyl]piperidine (4b)**

The mesylate derivative **3b** (46.37g, 0.175 moles) was added to a mixture of toluene (500.0mL), and 3,4-methylenedioxy phenol (24.6g, 0.178 moles) and powdered potassium carbonate (60.6g, 0.439 moles) and refluxed for 20-24 h. Water (1.0L) was added to the cooled reaction mass. The organic layer was separated and the aqueous layer was extracted with toluene (250.0mL). Both the combined organic layer was washed with 10% sodium hydroxide solution (250.0mLx 2), brine (250.0mLx 2). The organic layer was evaporated in *vacuo* to give the title compound **4b** (52.1g, 97%) as brown colored oil, IR (νmax, KBr): 3439, 2948, 2888, 2792, 1499, 1245, 1185, 1033, 787cm^-1^,^1^H NMR (CDCl_3_) : δ 6.7 (1H, d, J 9 Hz, H-5’), 6.5 (1H, d, J 3 Hz, H-2’), 6.3 (1H, dd, J 9.0, 3.0 Hz H-6’), 5.85 (2H, s, O_2_CH_2_), 4.18-4.0 (1H, m, H-3a), 3.98-3.7 (5H, m, H-3a, OCH_2_CH_2_O), 3.0-2.8 (2H, m, 2 xH-2), 2.7-2.5 (1H, m, H-6), 2.4-2.2 (5H, m, N-CH_3_, H-6, H-3), 1.8-1.6 (2H, m, 2 xH-5), ^13^C NMR (CDCl_3_): δ 33.99 (C-5), 43.39 (C-3), 45.68 (N-CH_3_), 53.18 (C-2), 56.35 (C-6), 64.34 (OCH_2_CH_2_O), 66.33 (CH_2_O), 97.90 (C-5’), 100.77 (O_2_CH_2_), 105.56 (C-2’), 107.38 (C-6’), 107.60 (C-4), 141.23 (C-1’), 147.90 (C-4’), 154.30 (C-3’), MS (m/z):307(M^+^), 170, 55, 99.

**3-Benzyl-1,2,3,4-tetrahydro[1,3]-dioxolo-[6,7]-5H-[1]benzopyrano[3,4-C]pyridine (5a)**

To the condensed product **4a** (61.28g, 0.16 moles), dilute sulphuric acid (360 mL H_2_SO_4_ in 600 mL water) was added slowly at room temperature and stirred for 24-30 h at the same temperature. After completion of the reaction, basified with 10 N sodium hydroxide solution to pH 9-10 and extracted with toluene (300.0mL x 3). The combined organic layer was washed with water and dried over sodium sulphate. The solvent was evaporated in *vacuo* to give the crude product, which was purified by column chromatography to give the title compound **5a** (25.7g, 50%) as light yellow solid, mp : 109-110 ^0^C (HCl salt mp: 199-202 ^0^C), IR (νmax, KBr): 3429, 1554, 1487, 1407, 1158, 1033, 654 cm^-1^ ,^1^H NMR (CDCl_3_): δ 7.4-7.2 (5H, br s, Ph), 6.6 (1H, s, H-7), 6.4 (1H, s, H-10), 5.9 (2H, s, O_2_CH_2_), 4.4 (2H, s, 2 x H-5), 3.7 (2H, s, CH_2_Ph), 3.0 (2H, s, 2 x H-4), 2.8 (2H, t, J 6.0 Hz, 2 x H-2), 2.4 (2H, br s, 2 xH-1), ^13^C NMR (CDCl_3_): δ 24.64 (C-1), 49.69 (C-2), 52.41 (C-4), 62.50 (N-CH_2_), 66.65 (C-5), 98.25 (C-10), 100.80 (O_2_CH_2_), 102.01 (C-7), 116.78 (C-10a), 122.72 (C-10b), 123.34 (C-4a), 127.07 (C-4’), 128.19 (C-2’,C-6’), 128.95 (C-3’,C-5’), 137.94 (C-1’), 141.77 (C-6a), 146.59 (C-9), 148.33 (C-8) MS (m/z) : 321 (M^+^) 246, 230, 202, 115

**3-Methyl-1,2,3,4-tetrahydro[1,3]-dioxolo-[6,7]-5H-[1]benzopyrano[3,4-C]pyridine (5b)**

To the condensed product **4b** (49.1g, 0.16 moles), dilute sulphuric acid (360 mL H_2_SO_4_ in 600 mL water) was added slowly at room temperature and stirred for 24-30 h at the same temperature. After completion of the reaction, basified with 10 N sodium hydroxide solution to pH 9-10 and extracted with toluene (300.0mL x 3). The combined organic layer was washed with water and dried over sodium sulphate. The solvent was evaporated in *vacuo* to give the crude product, which was purified by column chromatography to give the title compound **5b** (18.8 g, 48%) as off white solid, mp : 89-91 °C,IR (νmax, KBr):3427, 2941, 2795, 1484, 1276, 1161, 1034, 754 cm^-1^, ^1^H NMR (CDCl_3_): δ 6.6 (1H, s, H-7), 6.4 (1H, s, H-10), 5.9 (2H, s, O_2_CH_2_), 4.55 (2H, s, 2x H-5), 2.9 (2H, s, 2x H-4), 2.7 (2H, t, J 5.0 Hz, 2 x H-2), 2.37 (5H, br s, N-CH_3_, 2x H-1), ^13^C NMR (CDCl_3_): δ 24.78 (C-1), 45.64 (N-Me), 51.97 (C-2), 54.32 (C-4), 66.71 (C-5), 98.35 (C-10), 100.92 (O_2_CH_2_), 102.12 (C-7), 116.88 (C-10a), 122.75 (C-4a), 123.15 (C-10b), 141.90 (C-6a), 146.71 (C-9), 148.46 (C-8) MS (m/z) : 245 (M^+^) 202, 175, 115, 108, 91.

**1,2,3,4-Tetrahydro [1,3]-dioxolo-[6,7]-5H-[1] benzopyrano [3,4-C] pyridine (6) by Method I**

2,2,2-trichloroethylchloroformate (1.91g, 0.009moles) in 1,2-dichloroethane (10mL) was slowly added to a solution of the cyclised compound **5b** (2.247g, 0.009 mole) in 1,2-dichloroethane (40mL) and refluxed for 12h. The reaction mixture was washed with water (100mLx2) and evaporated the solvent in *vacuo*. The residue was dissolved in acetic acid (20mL) followed by addition of zinc dust (0.73g, 0.01moles). The mixture was heated to reflux at 80-90 ^0^C for 4hrs. Water (100mL) was added to the cooled reaction mass and extracted with 1,2-dichloroethane (50mLx3) followed by the water (100mLx2) washings to the combined organic layer. Then the combined aqueous layer was basified with sodium carbonate solution to pH 8.0-8.5 and extracted with dichloromethane (50mLx3). The solvent was evaporated in *vacuo* to give the crude product, which was purified by using ethyl acetate and diethyl ether to yield the title compound **6**(0.65g, 40%) as light yellow solid, mp : 123-125 ^0^C, IR (νmax, neat):3019, 2926, 1624, 1502, 1484, 1216, 1162, 757cm^-1^,^1^H NMR (CD_3_OD):δ6.75 (1H, s, H-7), 6.4 (1H, s, H-10), 5.95 (2H, s, O_2_CH_2_), 4.5 (2H, s, 2 xH-5), 3.7 (2H, s, 2x H-4), 3.45 (2H, t, J 6.0 Hz, 2x H-2), 2.65 (2H, br s, 2 xH-1); ^13^C NMR (CD_3_OD): δ 23.55 (C-1), 42.97 (C-2), 44.19 (C-4), 67.20 (C-5), 99.16 (C-10), 102.37 (O_2_CH_2_), 103.05 (C-7), 117.52 (C-10a), 122.68 (C-10b), 124.87 (C-4a), 143.42 (C-6a), 148.52 (C-9), 149.93 (C-8) MS (m/z) 231 (M^+^),203.

**1,2,3,4-Tetrahydro [1,3]-dioxolo-[6,7]-5H-[1] benzopyrano [3,4-C] pyridine (6) by Method II**

1-Chloroethyl chloroformate (1.7g, 0.012 moles) was added to a solution of the cyclised compound **5b** (0.924g, 0.004moles) in dichloromethane (10mL) and stirred for 4 h. After 4 h, 50% of the solvent was concentrated in vacuo and added methanol (5mL) followed by refluxing for 6 h. The solvent was removed and added water (100mL). The aqueous layer was basified with sodium carbonate solution to pH 8.0-8.5 and extracted with dichloromethane (50mLx3). The solvent was evaporated in *vacuo* and the residue was taken for column purification to yield the title compound **6** (0.13g, 20%) as light yellow solid, mp : 123-125 ^0^C and matches with the above spectral data of VI.

# Hydrogenation of 5a

10% Palladium on charcoal (5.2 g) was added to a clear solution of N- benzyl substituted cyclised compound **5a** (13 g, 0.04 moles) in methanol (200 mL) and 1 N hydrochloric acid (10mL) and stirred under hydrogen pressure of 40-50 psi at room temperature for 40-45 h. After completion of the starting material 5a in the reaction mass, the catalyst was filtered and the mother liquor was concentrated to give the crude hydrochloride salt. The crude compound was taken in chloroform (65mL) and basified with 1N sodium hydroxide solution to pH 8.0-8.5. The aqueous layer was separated and again extracted with chloroform (65 mL). The combined organic layer was washed with water (65mL) and dried over sodium sulphate. The solvent was removed in *vacuo* to give a gummy mass. Isopropyl alcohol (10.0 mL) was added to the gummy mass and the solid obtained was filtered to give the title compound **7** (6.9 g, 63%) as a white solid. [To isolate **6** or **8**, the hydrogen bubbling was done for 6-7h and the reaction mixture was worked up as mentioned above to get a mixture of free base product (**6, 7, 8**) and this was purified by column chromatography to get the individual component]

**(+) cis 1-Benzyl 1,3,4,4a,5,10b-hexahydro[1,3]-dioxolo-[6,7]-2H-[1]-benzopyrano-[3,4-C]-pyridine (8)**

Light yellow oil, IR (νmax,KBr): 3025, 2830, 2866, 2804, 1480, 1152, 1031, 734cm^-1^ ,^1^H NMR(CDCl_3_): δ7.3 (5H, br s, Ph), 6.58 (1H, s, H-7), 6.38 (1H, s, H-10), 5.87 (2H, s, O_2_CH_2_), 4.4-4.2 (1H, dd, J 10.0, 9.0 Hz,H-5), 4.1-3.9 (1H, dd, J 10.0, 3.0 Hz,H-5), 3.4 (1 H, d, J 13.0 Hz, CH_a_H_b_Ph), 3.3 (1 H, d, J 13.0 Hz, CH_a_H_b_Ph), 2.8-2.65 (2H, m, 2xH-4), 2.62 –2.4 (2H, m, 2xH-2), 2.36-2.02 (2H, m, H-4a,H-10b), 2.0-1.8 (2H, m, 2xH-1), ^13^C NMR (CDCl_3_): δ 31.4 (C-1), 32.6 (C-4a), 33.7 (C-10b), 52.5 (C-2), 54.1 (C-4), 63.1 (N-CH_2_), 66.7 (C-5), 98.31 (C-7), 100.6 (O_2_CH_2_), 107.2 (C-10), 117.3 (C-10a), 126.79 (C-4’), 128.04 (C-3’,C-5’), 128.59 (C-2’,C-6’), 138.53 (C-1’), 141.1 (C-9), 146.34 (C-6a), 148.54 (C-8), MS(m/z):323 (M^+^), 232, 176, 146, 91.

**(+) cis 1,3,4,4a,5,10b-Hexahydro[1,3]-dioxolo-[6,7]-2H-[1]-benzopyrano-[3,4-C]-pyridine (7)**

White solid, mp : 106-109 ^0^C (mp of HCl salt of VII: 248-51 ^0^C), IR (νmax, KBr): 3429, 1554, 1487, 1407, 1159, 1033, 930, 654 cm^-1^, ^1^H NMR (CD_3_OD): δ 6.7 (1H, s, H-7), 6.39 (1H, s, H-10), 5.82 (2H, s, O_2_CH_2_), 4.10-3.95 (2H, ddd, , J 11.0, 6.0, 3.0 Hz, 2 x H-5), 3.30 (1H, br s, NH), 3.23-3.03 (2H, ddd, J 8.0, 4.0, 2.0 Hz, 2xH-4), 3.02-2.88 (2H, m, 2xH-2), 2.78-2.68 (1H, ddd, J 10.0, 9.2, 3.2 Hz, H-10b), 2.28-2.21(1H, m, H-4a), 2.18-1.94 (2H, m, 2xH-1); ^13^C NMR (CD_3_OD): δ 26.89 (C-1), 30.98 (C-4a), 31.84 (C-10b) 41.14 (C-2), 43.0 (C-4), 66.79 (C-5), 98.88 (C-7), 101.8 (O_2_CH_2_), 106.8 (C-10), 114.2 (C-10a), 143.12 (C-9), 147.95 (C-6a), 149.56 (C-8), MS (m/z): 233(M^+^), 203, 189, 175, 96.

**(-)Cis-1,3,4,4a,5,10b-Hexahydro-[1,3[-dioxolo-[6,7]-2H-[1]-benzopyrano-[3,4-C] pyridine (7a)**

D(-) Mandelic acid (6.5 g, 0.042 moles) in methanol (20 mL) was added to a solution of **7** (9.0 g, 0.038 moles) in methanol (80 mL). This solution was stirred at 20-25 ^0^C for 12 h and the solid separated was filtered and washed with pre-cooled methanol (5 mL) to give the mandalate salt of **7a**, mp.180-186 ^0^C. The free base was regenerated from the mandalate salt using 10% sodium hydroxide solution to yield the (-) cis enantiomer, 2.5g ( **7a** ) as a white colored crystalline solid,mp.106-109 ^0^C, [α]_D_^25^:(-) 34.93 (C 1.0,MeOH) [Chiral HPLC purity:99.5%, Chiral HPLC conditions: Column: Chiralcel OD H, 150mm, Mobile phase: Hexane: Isopropyl alcohol: TFA(80:20:0.1), Flow rate: 1.0Ml/minute, λ: 215nm.].

**(+)Cis-1,3,4,4a,5,10b-Hexahydro-[1,3]-dioxolo-[6,7]-2H-[1]-benzopyrano-[3,4-C] pyridine (7b)**

The filtrate and the washings of 7a was concentrated and isopropyl alcohol (20 ml) was added to it and stirred at –5 to 0 ^0^C for 12 h, the solid separated was filtered and washed with methanol (2x5ml) followed by pre-cooled isopropyl alcohol (30 ml) to give the mandalate salt of **7b**,mp: 184-87 ^0^C.The free base was regenerated using 10% sodium hydroxide solution to yield the (+) cis enantiomer, 2.2g (**7b**) as a white colored crystalline solid, mp : 106-109 ^0^C , [α]_D_^25^: (+) 34.72 (C 1.0,MeOH) (Chiral HPLC purity:99%, HPLC condition same as mentioned in 7a)

**X-ray crystallographic analysis of mandalate salt of 7b :**

A well shaped monoclinic crystal of mandalate salt of **7b** was obtained by recrystallisation from chloroform-isopropyl alcohol mixture C_13_H_16_NO_3_.C_8_H_7_O_3_.1.5 H_2_O(412.44); space group C_2_, a = 23.126 (2) Å, b = 6.065 (1) Å, c = 15.256(2)Å, β = 110.57(1), V = 2003.3(9) Å, crystal dimensions 0.5 x 0.3 x 0.2 mm, Z = 4, d_calcd_ = 1.37 g / cm^3^, F(000) = 876e, Rigaku AFC7S diffractometer, Cu Kα = radiation (λ = 1.5418A), T = 25 °C. Data were corrected for Lorentz and polarization effects as well as for absorption [empirical, absorption coefficient = 8.73 cm^-1^]. 2665 reflections measure, 2027, [R(int) = 0.053] unique reflections. Non-H atoms were refined with anisotropic displacement parameters. Hydrogen atoms connected to oxygen and nitrogen atoms were located from a different Fourier map and refined isotropically. Rest of the hydrogen atoms were calculated in idealized geometry and included with isotropic contributions. Refined parameters (218) WR^2^ (unique data) = 0.0917, R^1^ [FO > 2σ (Fo)] – 0.0439, WR^2^ = [Σw(Fo^2^-Fc^2^)^2^]/Σw(Fo^2^)^2^]^1/2^, R1 = Σ(|| Fo | - | Fc ||, w= q/2σ^2^(Fo^2^) + (ap^2^) + bp, p – (Fo^2^ + 2Fc^2^)/^3^; a = 0.0241, b = 057051.
